# Physiological and Nitrogen Balance Responses of Adult Dogs Fed Diets Containing Different Crude Protein Levels

**DOI:** 10.3390/ani16213436

**Published:** 2026-10-30

**Authors:** Woo-Do Lee, Hyun-Woo Cho, Min Young Lee, Sang-Yeob Lee, Han Tae Bang, Ju Lan Chun, Seyeon Chang, Won Yong Jung, Kyoung-Min So

**Affiliations:** Animal Welfare Division, National Institute of Animal Science, Rural Development Administration, Wanju 55365, Republic of Korea; woodo92@korea.kr (W.-D.L.); jhwoo3856@korea.kr (H.-W.C.); mylee1231@korea.kr (M.Y.L.); sangnext@korea.kr (S.-Y.L.); banght80@korea.kr (H.T.B.); julanchun@korea.kr (J.L.C.); csy0127@korea.kr (S.C.); jwy95@korea.kr (W.Y.J.)

**Keywords:** adult dog, crude protein, nitrogen balance, apparent total tract digestibility, indoxyl sulfate

## Abstract

This nonrandomized sequential study evaluated nitrogen (N) balance, apparent nutrient digestibility, serum biochemistry, and indoxyl sulfate (IS)-related outcomes in ten healthy adult Beagles. Dogs received the crude protein (CP) 30% formulation for four weeks followed by the CP 18% formulation for four weeks; urine and feces were quantitatively collected during four consecutive days in each period. Analyzed dietary CP concentrations were 31.36% and 19.20% of dry matter. Daily urinary N excretion was lower in the second period (5.60 ± 0.35 vs. 3.34 ± 0.15 g/day; false discovery rate (FDR)-adjusted *q* < 0.001), as was calculated N balance (2.52 ± 0.39 vs. 1.04 ± 0.14 g N/day; paired *p* = 0.0022). The N retention percentages were 28.78 ± 4.45% and 20.66 ± 2.81%, respectively (*p* = 0.077; *q* = 0.132), and were not demonstrated to be equivalent. Apparent total tract digestibility of CP and most analyzed amino acids was lower in the second period. Exploratory daily urinary IS excretion was 3.08 ± 0.35 and 2.59 ± 0.36 mg/day, respectively (*p* = 0.394; *q* = 0.394). The analyzed indispensable amino acid concentrations of both diets met the dietary nutrient profile benchmarks evaluated in this study. However, the formulations differed in ingredients and predicted daily energy intake, and period was completely confounded with feeding order. These observations cannot establish an isolated effect of dietary protein, actual long-term body protein retention, or nutritional equivalence.

## Simple Summary

Protein quality and nitrogen disposal are important when comparing dog foods with different protein concentrations. Ten healthy adult Beagles were fed a higher-protein experimental formulation for four weeks and then a lower-protein formulation for four weeks. Urine and feces were collected for four consecutive days in each period. During the second period, urinary nitrogen excretion and calculated nitrogen balance were lower, as were the apparent digestibilities of crude protein and most amino acids. Both periods showed positive calculated nitrogen balance, but this does not prove long-term protein adequacy or body protein retention. The fixed feeding sequence and differences in ingredient composition and energy intake prevent attribution of the observed changes to protein concentration alone.

## 1. Introduction

Dietary nutrients, including protein, fat, carbohydrates, and minerals, should be appropriately balanced in complete pet foods to support normal physiological function and long-term health in companion animals [1,2,3]. Among these nutrients, protein is of particular interest because it supplies essential amino acids required for tissue maintenance, metabolic regulation, and the synthesis of enzymes and hormones. The crude protein (CP) content of commercially available dog foods varies widely—ranging from approximately 18% to over 60%—resulting in significant differences in nutrient composition (including amino acid profiles) and digestibility [2,3,4,5]. Variations in nutrient content may reflect consumer preferences and can contribute to excessive nutrient intake, feed wastage, and obesity [1,4]. Therefore, further evaluation of appropriate dietary protein levels is warranted to support canine health [6,7,8,9].

Dietary protein should be supplied at a level that meets dogs’ physiological requirements without unnecessary excess [10]. Studies in other species, including poultry, pigs, and dairy cattle, have reported that insufficient protein intake may limit nitrogen (N) availability and the supply of essential amino acids, whereas protein intake exceeding physiological requirements may increase amino acid catabolism and N excretion [11,12,13]. Accordingly, reducing dietary CP while maintaining an adequate supply of essential amino acids may be a potential strategy for improving protein utilization efficiency and reducing N excretion [14]. However, this strategy requires dietary CP and essential amino acids to be supplied at appropriate levels to meet dogs’ nutritional requirements [15]. From a nutritional sustainability perspective, optimizing dietary protein levels is also important for promoting the efficient use of feed resources while maintaining nutritional adequacy [1]. Nevertheless, compared with other animal species, research evaluating the effects of dietary protein levels on physiological responses and N utilization efficiency in dogs remains relatively limited [10,16]. Therefore, further research is needed to determine dietary CP levels that can improve protein utilization efficiency while maintaining nutritional adequacy in dogs.

In livestock nutrition, reducing dietary CP to levels that more closely match physiological requirements has been investigated as a strategy to reduce N excretion and environmental burden while maintaining productive performance [17,18,19]. A similar question is relevant to companion animal nutrition because commercial diets may provide substantially more protein than the minimum recommended level for adult maintenance [4]. Particularly in South Korea, where nutritional guidelines for complete pet foods are being incorporated into the regulatory framework under the Feed Management Act, empirical data are needed to support the development and refinement of domestic nutritional standards [5].

The Association of American Feed Control Officials (AAFCO) and the National Institute of Animal Science (NIAS) in Korea recommend a minimum CP concentration of 18% dry matter (DM) for the maintenance of healthy adult dogs [20,21]. Based on an energy density of 4000 kcal metabolizable energy (ME)/kg DM, this concentration is equivalent to 45 g CP/1000 kcal ME. However, empirical evidence regarding the physiological and nutritional responses of adult dogs fed diets formulated near this minimum recommended level remains limited [4,22]. Therefore, the present study provides an integrated within-dog descriptive assessment of urinary and fecal N losses, calculated N balance, nutrient and amino acid apparent digestibility, serum biochemistry, and IS-related outcomes during two fixed-order feeding periods. It was not designed to demonstrate long-term protein adequacy or equivalence. Because the formulations were administered in the same order, feeding period and formulation effects cannot be separated.

## 2. Materials and Methods

The protocol for this experiment was reviewed and approved by the Institutional Animal Care and Welfare Committee of the NIAS, Rural Development Administration, Republic of Korea (NIAS2025-0013).

### 2.1. Experimental Diets and Nutrient Composition

In this study, two experimental dry diets were prepared to contain 30% and 18% CP on a dry matter (DM) basis, respectively, while meeting the nutrient requirements for adult dogs at maintenance [21]. The preparation method was based on the procedure described by Seo et al. [23]. Briefly, all ingredients, except for lard, were commercially available powdered products. The dry ingredients were mixed at 500 rpm for 10 min using a food paste mixer (Mixer, Sung-il, Seoul, Republic of Korea); subsequently, lard and water were added, and the mixture was kneaded at 1500 rpm for 20 min. The dough was divided into portions approximately 10 cm in diameter and steamed at ≥100 °C for 40 min using a steamer (Rice Cake Maker, Dahan, Seoul, Republic of Korea). The steamed dough was then formed into cylindrical pellets (10 mm in diameter and 15 mm in length) using a noodle-making machine (Noodle Maker, Yusung, Daegu, Republic of Korea). The pellets were dried at 70 °C for 1 h in an oven (Dry Oven, Shiniltech, Jeonju, Republic of Korea) and stored at −20 °C until feeding. One day prior to feeding, the diet was removed from the freezer and allowed to equilibrate to room temperature. No palatability enhancers were added to the diet. The ingredient and analyzed nutrient compositions of the experimental diets are presented in Table 1.

Proximate analysis showed that the experimental diets contained 31.36% and 19.20% CP on a DM basis, respectively, slightly exceeding the formulated targets of 30% and 18%. This discrepancy is consistent with previous reports indicating that the analyzed nutrient composition of pet foods may differ from formulated or guaranteed values [24]. The proximate and amino acid compositions were determined using official Korean standard feed analytical methods; amino acid testing was performed by an external accredited testing laboratory. The NFE was calculated on a DM basis from the measured proximate fractions as:

NFE (% DM) = 100 − [CP (% DM) + EE (% DM) + CF (% DM) + ash (% DM)]

Gross energy (GE), predicted energy digestibility, digestible energy (DE), and metabolizable energy (ME) were estimated from the analyzed proximate composition using the equations described in the Korean Feeding Standard for Dog and Cat [21]:

GE (kcal/100 g DM) = 5.7 × CP + 9.4 × EE + 4.1 × (NFE + CF)

Energy digestibility (%) = 91.2 − 1.43 × CF

DE (kcal/100 g DM) = GE × Energy digestibility/100

ME (kcal/100 g DM) = DE − 1.04 × CP

In these equations, CP, ether extract (EE), crude fiber (CF), NFE, and ash are entered numerically as g/100 g DM; the factor 1.04 is the urinary energy correction (kcal/g CP). The results in kcal/100 g DM were multiplied by 10 to report kcal/kg DM. For example, using the unrounded CP 30% diet values, GE = 486.473 and DE = 443.576 kcal/100 g DM; ME = 443.576 − (1.04 × 31.356) = 410.965 kcal/100 g DM or approximately 4109.7 kcal/kg DM. The corresponding predicted CP 18% dietary ME was 4096.7 kcal/kg DM. Dietary GE, DE, and ME were calculated, not directly determined by calorimetry or urinary energy measurement. Measured moisture contents were 20.75% and 27.98% as fed; combined with complete consumption of fixed rations, these correspond to predicted daily ME intakes of approximately 716.5 and 669.8 kcal, respectively, which were not energy-matched.

The analyzed indispensable amino acid profiles were obtained by an external accredited testing laboratory using official standard feed analytical methods. Table 2 summarizes the dietary concentrations as % DM and g/1000 kcal predicted ME alongside the adult maintenance comparison values assembled from NIAS, AAFCO, NRC recommended allowances, and FEDIAF [20,21,25,26]. Both formulations met all of the specific nutrient profile reference values used in that comparison. This composition based assessment does not independently establish adequate daily intake for every possible energy requirement or prove long-term physiological sufficiency.

### 2.2. Animals and Experimental Design

Ten clinically healthy adult Beagle dogs (five females and five males; mean age, 2.33 ± 0.01 years [reported range, 2.32–2.34 years]; mean body weight [BW], 10.2 ± 2.4 kg; mean body condition score [BCS], 5.2 ± 1.23) were included under veterinary supervision. All dogs were neutered. Before the study, they had consumed a commercial complete dog food containing approximately 30% CP (according to the product guaranteed analysis) for approximately three months. Dogs were housed separately in individual indoor rooms measuring approximately 1.70 × 2.10 m per dog, at 25 ± 4.4 °C and 75 ± 7.2% relative humidity. Windows provided predominantly natural light, supplemented by artificial lighting. Water was freely available. During regular housing, access to an outdoor exercise area was provided for approximately 3 h/day, allowing voluntary activity; this does not imply three hours of continuous exercise by each animal.

This was a nonrandomized within-dog sequential feeding study. Every dog received the CP 30% formulation for four weeks, followed without washout by the CP 18% formulation for four weeks. Each period included three weeks in ordinary individual housing and a final week involving metabolic cage collection (cage size: width 110 cm, depth 72 cm, height 79 cm). A reference maintenance energy estimate of 130 kcal × kg BW^0.75^/day was considered during protocol design [27], but daily rations were not individually adjusted for BW. Each dog received 220 g/day of the CP 30% diet or 227 g/day of the CP 18% diet, offered once daily. Individual consumption was monitored, all feed was consumed every day, and no refusals occurred. Dietary nitrogen concentrations measured by Kjeldahl analysis were 3.98% and 2.21% on an as-fed basis; thus, N intake was 8.756 and 5.017 g N/day, respectively (displayed as 8.8 and 5.0 after rounding, respectively). No formal a priori sample size or power calculation was performed. Sex by period responses were not separately analyzed.

### 2.3. Measurements and Sample Collection

The BCS was assessed visually and by palpation using the 9-point system of Laflamme [28], with evaluations by two trained veterinarians. Fecal consistency was evaluated daily by trained animal care personnel and research staff using the predefined 5-point scale of Middelbos et al. [29] (1, hard and dry; 2, firm and formed; 3, soft but formed; 4, soft and unformed; 5, watery). Scores of 2–3 were considered desirable [30]. Fecal scores were recorded daily, and the mean fecal score for each dog within each dietary period was used for statistical analysis. The dog, rather than a technical assessment or rater, was treated as the statistical unit. Inter-rater reliability was not formally assessed.

Blood samples were obtained by jugular venipuncture after an approximately 12 h fast on the final day of each feeding period. Ethylenediaminetetraacetic acid (EDTA) tubes and serum separator tubes were used for hematology and serum biochemistry, respectively. Serum was separated promptly by centrifugation at 3000× *g* for 15 min at 4 °C (VISION SCIENTIFIC Co., Ltd., Daejeon, Republic of Korea). Serum biochemical measurements, including urea and ammonia, were performed immediately after separation using a cobas c 111 automated chemistry analyzer (Roche Diagnostics, Indianapolis, IN, USA) and the manufacturer’s reagents and instructions. Remaining separate aliquots intended for later assays were stored at −80 °C. The ammonia assay used a glutamate dehydrogenase-based reaction; the study measured ammonia in serum, although the manufacturer’s NH3L documentation specifies EDTA plasma as the validated specimen, an analytical limitation acknowledged below.

During the final week of each feeding period, dogs were housed individually in metabolic cages from Monday to Friday. Urine and feces were separated using slatted cage floors and plastic collection trays. Urine accumulated in unacidified plastic trays for approximately 24 h and was recovered once daily at about the same time over four successive collection intervals. The mass of recovered urine was measured separately for each dog; no spillage, leakage, or incomplete physical recovery was observed. Urinary pH, specific gravity, and total N were assessed immediately after daily recovery without preservatives or intermediate storage. Urinary N was measured using the Kjeldahl method. Feces were collected and weighed daily; daily aliquots for fecal N were dried at 60 °C for three days and homogenized. Separate individual four-day fecal composites for apparent nutrient digestibility were stored at −80 °C and subsequently freeze-dried and ground. On the final day, the entire approximately 24 h urine collection was thoroughly mixed before an aliquot was collected and stored at −80 °C for urinary IS and creatinine analyses. Urinary N volatilization before collection was not measured despite the absence of physical spills.

### 2.4. Laboratory Analyses

Complete blood counts were analyzed using an automated veterinary hematology analyzer (BC-5000VET; Mindray, Shenzhen, China). Serum biochemical parameters, including urea and ammonia, were measured immediately after serum separation on an automated clinical chemistry analyzer (cobas c 111; Roche Diagnostics), following the respective manufacturer’s instructions. The ammonia assay was based on glutamate dehydrogenase; this study used serum rather than the manufacturer’s recommended EDTA plasma matrix.

Serum and urinary indoxyl sulfate (IS) concentrations were measured using a canine-specific competitive ELISA kit (MyBioSource, Cat. No. MBS737914, San Diego, CA, USA) according to the manufacturer’s procedure. Serum and urine were tested undiluted in triplicate technical wells, and absorbance was read at 450 nm. Sample concentrations were derived from calibration standards expressed in μg/mL. Urinary creatinine was measured using an automated chemistry analyzer (AU480; Beckman Coulter, Brea, CA, USA); the IS-to-creatinine ratio was expressed in μg/mg creatinine [31]. For each feeding period, estimated daily urinary IS excretion (mg/day) was calculated from the final day representative 24 h IS concentration (μg/mL) multiplied by corresponding urine volume (mL/day), divided by 1000. Because urine was weighed, volume was calculated as urine mass (g)/individual specific gravity (approximating water density as 1 g/mL). No independent urine matrix recovery or intra-assay CV data were available for reporting.

Dietary, urinary, and fecal N concentrations were determined using the Kjeldahl method with a Kjeltec 2300 nitrogen analyzer (FOSS Analytical, Hillerød, Denmark), as also described for nitrogen analysis using this instrument in a previous study [32]. Proximate and amino acid compositions of the diets were determined using official Korean standard feed analytical methods; dietary amino acids were tested by an external accredited testing laboratory. Dietary GE, DE, and ME were estimated from analyzed composition rather than directly measured. NFE was calculated by difference.

### 2.5. Nitrogen Balance and Apparent Total Tract Digestibility Calculations

Daily dietary N intake was calculated for each dog as the actual feed consumed multiplied by the analyzed as-fed dietary N fraction (3.98% for CP 30% and 2.21% for CP 18%). Daily urinary N excretion was obtained from measured urine output and Kjeldahl N concentration and daily fecal N excretion from fecal DM output and Kjeldahl N concentration on a DM basis. Calculated N balance was derived from the four-day collection period as follows; miscellaneous and integumentary N losses were not measured:

N balance (g N/day) = N intake − (urinary N excretion + fecal N excretion)

Total N excretion (g N/day) = urinary N excretion + fecal N excretion

BW-normalized N excretion = total N excretion/BW^0.75^

Total N excretion relative to intake (%) = 100 − N retention (%).

N retention (%) = (N balance/N intake) × 100

Apparent total tract digestibility (ATTD) of nutrients and amino acids was determined by the quantitative total fecal collection method [33]. Values were calculated for each dog using total dietary nutrient intake and fecal nutrient output during four collection days. Predicted dietary GE, DE, and ME were not represented as directly measured energy digestibility outcomes:

ATTD (%) = [(nutrient intake − measured fecal nutrient output)/nutrient intake] × 100

### 2.6. Statistical Analysis

Each dog was the experimental unit (*n* = 10). Statistical analyses were performed using paired two-sided *t*-tests for continuous endpoints in R version 4.2.3 and the Wilcoxon signed-rank test for ordinal BCS and fecal scores. Within-dog differences were examined using the Shapiro–Wilk test, with Wilcoxon comparisons used as sensitivity analyses where appropriate. The revised analysis treats calculated N balance as the main outcome; descriptive and exploratory secondary outcomes were adjusted using the Benjamini–Hochberg false discovery rate (FDR) within outcome families. The excretory/N-related secondary family comprises 12 tests, including added absolute and BW^0.75^-normalized total N excretion; the IS family comprises five outcomes, including new daily IS excretion. Deterministically assigned ration masses and algebraically complementary excretion/retention percentages were not treated as independent testing endpoints. Paired mean differences are CP 18% minus CP 30%, with two-sided 95% confidence intervals (CI). Significance was evaluated at *p* < 0.05 for the primary N balance analysis and FDR *q* < 0.05 for exploratory outcomes. Values are mean ± standard error of the mean (SEM) unless otherwise noted. Neither statistical non-significance nor profile value compliance establishes equivalence or long-term adequacy.

## 3. Results

### 3.1. Body Characteristics and Fecal Score

BW and BCS at weeks 0, 4, and 8 are presented in Table 3. BW did not change significantly within either feeding period. BCS did not change significantly during the CP 30% (*p* = 0.188) or CP 18% (*p* = 1.000) feeding period, as determined by the Wilcoxon signed-rank test.

**Figure 1:**
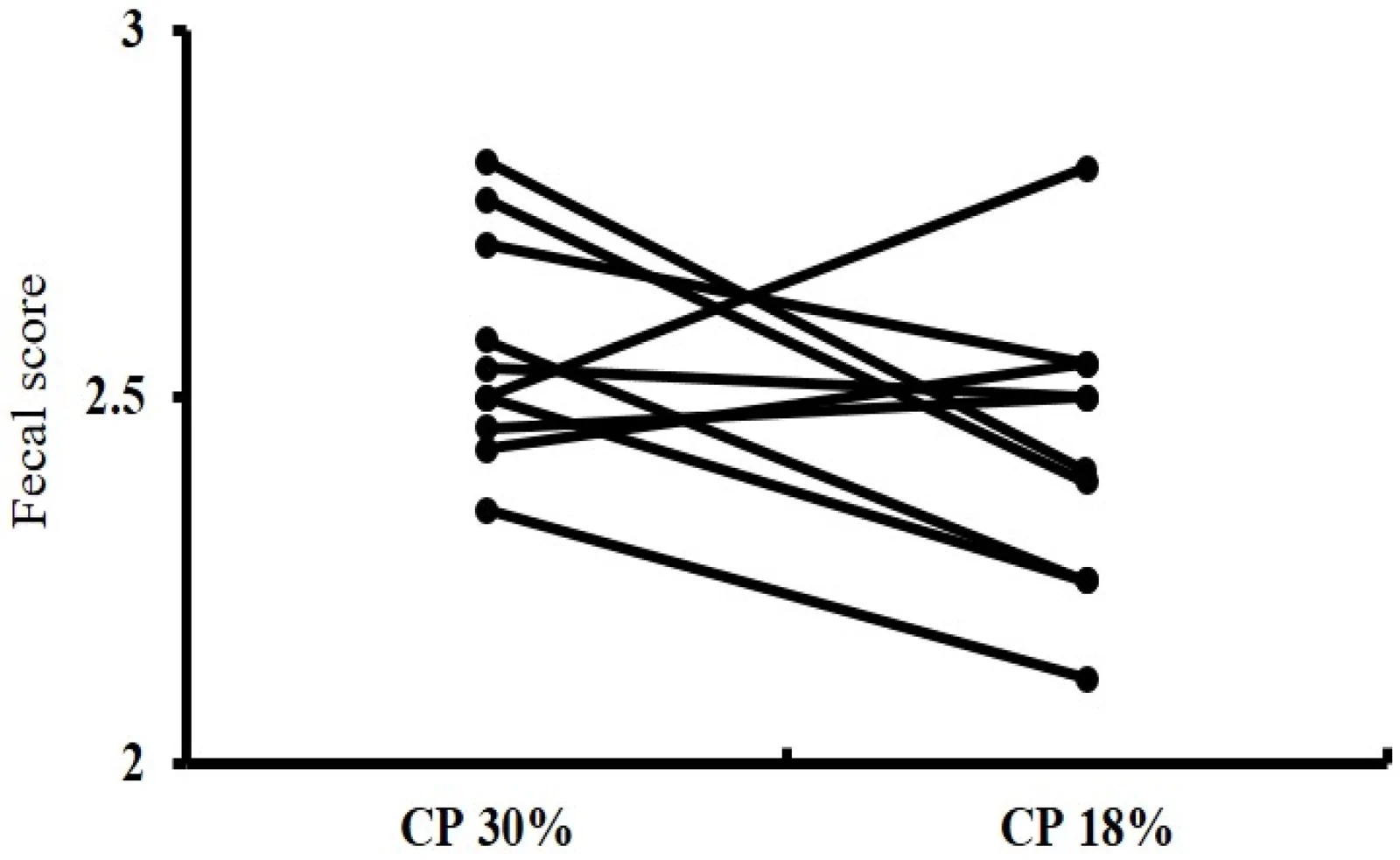
Individual paired fecal scores of adult dogs during the CP 30% and CP 18% dietary periods. Each point represents the mean fecal score for one dog within each dietary period, and lines connect observations from the same dog. Fecal scores were compared between dietary periods using the Wilcoxon signed-rank test (*p* = 0.131).

Mean fecal scores did not differ significantly between feeding periods (CP 30%, 2.565 ± 0.049; CP 18%, 2.430 ± 0.062; paired Wilcoxon *p* = 0.131; Figure 1).

### 3.2. Serum Biochemical Parameters

Serum urea and serum ammonia values were numerically lower during the CP 18% period (urea: 26.2 ± 0.97 vs. 17.3 ± 0.67 mg/dL; ammonia: 204.8 ± 10.27 vs. 84.5 ± 3.44 μg/dL; both *q* < 0.001). Ammonia was measured in serum although the reagent manufacturer validates EDTA plasma; the results therefore require cautious interpretation. LDH (*p* = 0.039; *q* = 0.166) and glucose (*p* = 0.020; *q* = 0.113) differed only in nominal comparisons, not after FDR adjustment. Serum total protein did not differ significantly (*p* = 0.074). Published canine serum biochemical reference ranges are provided for descriptive comparison in Table 4; they were not established on the study analyzer.

Hematological parameters are summarized in Supplementary Table S1. Mean corpuscular hemoglobin concentration (MCHC) was the only hematological parameter that differed significantly between feeding periods after FDR adjustment (*p* < 0.001, *q* < 0.001). Although eosinophil counts differed in the unadjusted comparison (*p* < 0.05), this difference was not significant after FDR adjustment (*q* = 0.293).

### 3.3. Nitrogen Balance and Indoxyl Sulfate Concentrations

The N and excretory outcomes are presented in Table 5. During the CP 18% period, water intake (paired *p* < 0.001; FDR *q* = 0.00095), urine output (*p* = 0.0034; *q* = 0.00683), urinary N excretion (*p* < 0.001; *q* < 0.001), and calculated total N excretion (*p* < 0.001; *q* < 0.001) were lower than during CP 30%. Total N excretion was 6.24 ± 0.39 versus 3.98 ± 0.14 g/day, and BW^0.75^-normalized output was 1.125 ± 0.095 versus 0.726 ± 0.048 g/kg BW^0.75^/day. Calculated N balance remained positive in both periods but was lower during CP 18% (2.52 ± 0.39 versus 1.04 ± 0.14 g/day; paired difference −1.48 g/day, 95% CI −2.28 to −0.69; *p* = 0.0022). The N retention percentage was numerically lower but not statistically significant (28.78 ± 4.45 vs. 20.66 ± 2.81%; paired difference −8.12 percentage points, 95% CI −17.33 to 1.09; *p* = 0.0772, *q* = 0.1323). Total N excretion constituted 71.22 ± 4.45% versus 79.34 ± 2.81% of N intake, respectively. This complementary percentage was not tested as an additional independent endpoint.

**Figure 2:**
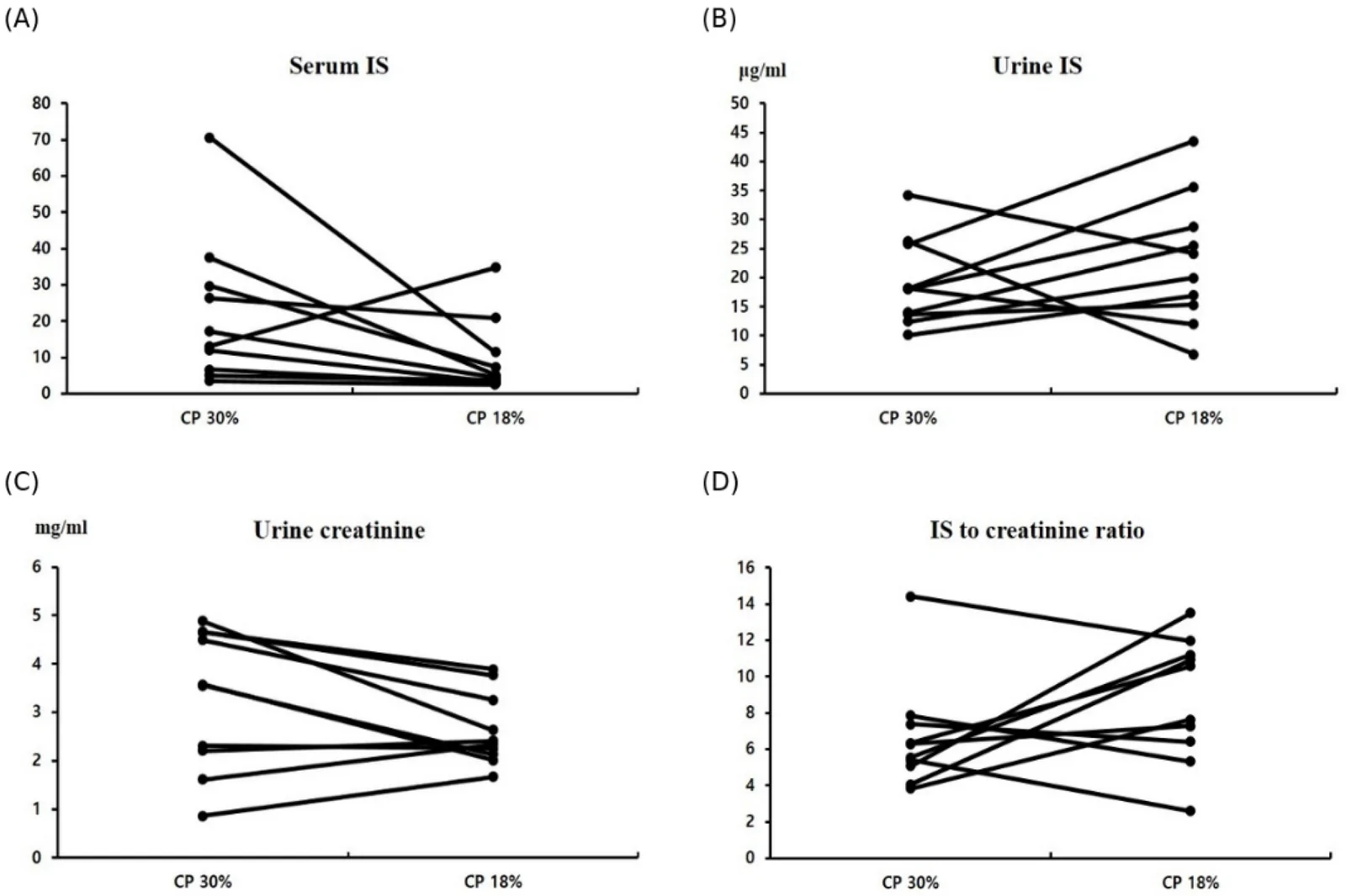
Within-dog paired serum and urinary indoxyl sulfate (IS)-related outcomes during the CP 30% and CP 18% feeding periods: (**A**) serum IS concentration, (**B**) urinary IS concentration, (**C**) urinary creatinine concentration, and (**D**) urinary IS to creatinine ratio. Lines connect measurements of the same dog (*n* = 10). Daily urinary IS excretion, calculated separately using representative 24 h urine samples and corresponding urine volume, is reported in Section 3 but is not depicted in this figure.

Serum and urinary IS-related measurements are shown in Figure 2. Serum IS values averaged 22.1 ± 6.46 and 9.6 ± 3.32 μg/mL during the CP 30% and CP 18% periods, respectively (paired difference −12.53 μg/mL, 95% CI −28.05 to 2.99; raw *p* = 0.101; revised five outcome IS family *q* ≈ 0.242). Estimated 24 h urinary IS excretion, computed from the representative final day sample and same day urine mass corrected by individual specific gravity, was 3.08 ± 0.35 and 2.59 ± 0.36 mg/day, respectively (paired difference −0.49 mg/day, 95% CI −1.72 to 0.74; raw *p* = 0.394; *q* ≈ 0.394). None of the five IS-related outcomes were statistically significant after FDR correction. The daily IS excretion values represent a derived exploratory endpoint.

### 3.4. Apparent Total Tract Digestibility

The ATTD values of measured nutrients and analyzed amino acids are shown in Table 6. The ATTD of CP was lower during the CP 18% period (85.7 ± 1.06%) than during the CP 30% period (92.1 ± 0.96%). Ash ATTD was also lower during the CP 18% period (43.4 ± 4.37%) than during the CP 30% period (60.4 ± 4.25%). DM, EE, and NFE ATTD values did not differ significantly after the stated comparisons; NFE means were 97.6738% and 97.7139%, both displayed as 97.7% after rounding. GE and ME digestibility rows were removed because energy losses had not been directly measured and the dietary GE/DE/ME concentrations were predicted rather than experimentally determined.

Most analyzed amino acids showed lower apparent total tract digestibility during the CP 18% period. Cystine digestibility showed no statistically significant difference. These apparent digestibility measures include endogenous and microbial fecal contributions and should not be interpreted as true digestibility or independent confirmation of dietary protein adequacy.

The ATTD values of selected nonessential amino acids are presented in Supplementary Table S2. The ATTD values of serine, glycine, and alanine were lower during the CP 18% period than during the CP 30% period (all *p* < 0.001). The ATTD values of aspartic acid, glutamic acid, and proline were also lower during the CP 18% period (all *p* < 0.05).

## 4. Discussion

This nonrandomized fixed-order feeding study provides a descriptive within-dog assessment of N intake, excretion, calculated balance, nutrient and amino acid apparent digestibility, and selected biochemical and IS-related outcomes. The second feeding period was associated with lower absolute N output and calculated N balance, but the formulations and periods were not separable experimentally. The results therefore cannot be attributed exclusively to CP concentration.

The present study evaluated the short-term physiological, N balance, and digestibility responses of healthy adult Beagle dogs to CP 30% and CP 18%. Overall, BW and fecal score did not differ significantly between CP 30% and CP 18%, and most mean hematological values were within the stated reference intervals. These findings suggest that CP 18% did not induce clinically apparent adverse responses during the short-term feeding period. However, N balance and the apparent total tract digestibility of CP and most amino acids were lower in CP 18% than in CP 30%. N retention was numerically lower in CP 18%, but the difference was not significant. Therefore, the present findings indicate that CP 18% supported short-term physiological stability and positive N balance in healthy adult dogs, although it did not provide the same magnitude of N balance or apparent protein and amino acid digestibility as CP 30%.

BW and fecal score are practical indicators for evaluating the short-term tolerance of dietary changes in dogs. In the present study, BW and fecal scores showed no statistically significant differences in the stated comparisons. Maintenance of BW in adult dogs is primarily influenced by energy intake, but sufficient dietary protein and amino acid supply are also required to support tissue maintenance and normal physiological function [1,34,35]. Fecal quality can be influenced by dietary protein level, protein digestibility, and the amount of undigested protein reaching the hindgut, where proteolytic fermentation may produce nitrogenous metabolites and affect fecal characteristics [13,36,37,38]. Furthermore, as all fecal scores in this study remained within an acceptable range, with no adverse changes observed, an 18% CP diet appears suitable under the short-term conditions examined [30]. However, the high digestibility of chicken breast powder may have influenced these results, and responses may vary depending on the protein source, overall dietary composition, and processing methods [15,39,40,41]. Therefore, further research is warranted to determine whether similar outcomes can be achieved using a wider range of protein sources.

In the present study, neither serum TP nor ALB concentrations differed significantly between the CP 30% and CP 18% feeding periods. Serum TP and ALB are representative indicators of body protein nutritional status and are commonly used to assess long-term protein sufficiency in healthy individuals [19,42]. Increased dietary protein intake generally increases the availability of digestible protein, resulting in elevated plasma protein concentrations [43]. Because circulating ALB concentrations decrease under conditions of inadequate dietary protein and energy intake or malnutrition [44], the absence of a significant difference in serum ALB concentrations between feeding periods does not necessarily establish the nutritional adequacy of either diet. Dogs fed the CP 30% diet exhibited higher blood GLU concentrations, which may be attributed to excess dietary protein and amino acids being converted to GLU via gluconeogenesis [43]. Blood urea and ammonia concentrations were lower during the CP 18% period than during the CP 30% period with decreasing dietary CP levels, reflecting a reduced N load [13,45]. As urea is a key indicator of protein metabolism and dietary amino acid utilization efficiency, lower blood urea concentrations may suggest improved protein utilization efficiency at lower CP levels [46]. However, most measured biochemical values remained within historically established reference intervals for healthy adult Beagles [15,47] and general canine populations [48,49,50,51,52]. Therefore, the observed changes did not indicate adverse effects on nutritional utilization or overall health status.

The clearest response to CP 18% was observed in N-related serum and urinary indices. Serum urea and ammonia concentrations were lower in CP 18% than in CP 30%. Because urea and ammonia are closely associated with amino acid catabolism and N disposal, these decreases likely reflect lower dietary N intake and reduced N load in CP 18% [13,45]. Although urinary N concentration did not differ between CP 30% and CP 18%, urinary N excretion was higher in CP 30%. This difference was likely related to the higher urine output and greater dietary N intake in CP 30%. In contrast, fecal N concentration was higher in CP 30%, but fecal N excretion did not differ between groups. These findings suggest that the difference in N output between diets was driven mainly by urinary N disposal rather than fecal N loss. However, the lower urinary N excretion in CP 18% should not be interpreted as evidence of improved protein utilization efficiency or renal protective effects.

Although urinary N excretion was lower in CP 18%, N balance was also lower in CP 18% than in CP 30%. This distinction is important because reduced N excretion alone does not necessarily indicate improved N utilization. N balance reflects the integrated outcome of N intake, digestion, absorption, metabolism, and excretion, and it remains a central indicator in dietary protein evaluation [53,54]. In the present study, the higher N balance in CP 30% can be explained by the substantially greater dietary N intake, even though urinary N excretion was also higher in this group. Conversely, CP 18% resulted in lower absolute N balance, but positive N balance was maintained. A positive calculated N balance was observed during CP 18%, but this result alone does not establish maintenance of body protein stores or equivalence with the CP 30% period.

The digestibility results help explain the lower N balance observed in CP 18%. The apparent total tract digestibilities of DM, EE, and NFE did not differ significantly between feeding periods. Energy digestibility was not directly measured in this study. In contrast, the apparent total tract digestibility of CP and most amino acids was lower in CP 18%. Nutrient digestibility in dogs can be affected by dietary CP concentration, protein source, ingredient composition, processing method, and animal-related factors [15,55,56]. The lower apparent digestibility of CP and amino acids in CP 18% may have contributed to the lower N balance observed in this group.

However, the lower apparent digestibility of CP and amino acids in CP 18% should not be interpreted as direct evidence of impaired intestinal absorption. Apparent total tract digestibility does not distinguish undigested dietary protein from endogenous N sources, including digestive enzymes, sloughed epithelial cells, microbial N, and other endogenous secretions [15,57]. When dietary protein intake is reduced, endogenous N losses may account for a greater proportion of total fecal N, thereby lowering apparent CP and amino acid digestibility values. Therefore, the lower apparent digestibility observed in CP 18% should be interpreted as an apparent digestibility response under lower protein intake conditions rather than as evidence of reduced absorptive capacity. This interpretation is consistent with the maintenance of positive N balance in CP 18%.

Serum and urinary IS concentrations did not differ significantly between CP 30% and CP 18%. The IS is a protein-bound uremic toxin derived from microbial metabolism of tryptophan to indole, followed by hepatic sulfation and renal excretion [58,59]. Because IS accumulation is strongly influenced by renal clearance, dietary effects may be less apparent in healthy animals (e.g., dogs and cats) with normal renal function than in animals with impaired kidney function [60,61,62]. In the present study, the absence of differences in serum IS, urinary IS, and creatinine-normalized urinary IS suggests that the difference between CP 30% and CP 18% was not sufficient to alter IS homeostasis under short-term conditions in healthy adult dogs. However, this result should not be interpreted as evidence of renal protection. Further studies are needed to determine whether dietary CP level affects IS production or clearance in dogs with altered renal function, different gut microbial profiles, or longer feeding durations.

Because the experimental period was short, the present findings should be interpreted as short-term physiological and nutritional responses rather than as evidence of long-term nutritional adequacy. Nevertheless, this study provides empirical evidence that CP 18% can maintain positive N balance, stable BW, acceptable fecal characteristics, and most hematological values within reference intervals in healthy adult dogs during a short-term feeding period. Further long-term and randomized studies are needed to confirm the nutritional adequacy of diets formulated near the minimum recommended CP level under different physiological and feeding conditions.

Additional limitations include the fixed CP 30%→CP 18% sequence without randomization, counterbalancing, or washout; prestudy familiarity with a commercial approximately 30%-CP diet; ingredient and moisture differences; predicted daily ME intakes differing by approximately 6.5%; ten dogs from a single breed without a prospective power calculation or sex by period evaluation; four-week interventions and four-day collection periods; and possible period/carryover effects. Urine was unacidified during approximately 24 h tray collection; volatile N loss was not quantified despite complete physical recovery. Miscellaneous and integumentary N losses and direct lean body mass/protein turnover measurements were unavailable. Dietary energy values were predicted, not determined by energy balance calorimetry, and ammonia was assayed in serum rather than the reagent manufacturer’s recommended plasma. Most secondary outcomes were exploratory, and a lack of statistical significance cannot establish equivalence. Although the dietary amino acid profiles met the evaluated nutrient density criteria, these data do not prove long-term physiological adequacy for every dog.

## 5. Conclusions

In this nonrandomized sequential study of healthy adult Beagles, the second, lower CP feeding period was associated with lower urinary and calculated total N excretion, lower calculated N balance, and lower apparent digestibility of crude protein and most amino acids. Calculated balance remained positive in both four-day collection periods but should not be interpreted as measured body protein accretion or evidence of long-term protein adequacy. The N retention percentage and exploratory daily urinary IS excretion were not significantly different after multiplicity adjustment, but equivalence was not established. Differences in ingredients, estimated energy intake, and fixed feeding order preclude an isolated causal interpretation of dietary CP concentration. Larger randomized feeding studies with longer follow-up and direct protein status measurements are needed.

## Figures and Tables

**Table 1:** Ingredient composition and analyzed nutrient composition of experimental diets containing 30% or 18% crude protein for adult dogs.

Items	Experimental Diet	
	CP 30%	CP 18%
	Ingredient composition, %	
Rice powder	43.8	56.0
Chicken breast powder	19.0	7.0
Egg yolk powder	10.0	10.0
Cabbage powder	2.0	2.0
Seaweed powder	1.6	1.6
Lard	1.5	1.5
Calcium carbonate	1.1	0.9
Calcium phosphate (mono)	1.4	1.4
Potassium citrate	0.9	0.9
Vitamin and mineral premix ^1^	0.5	0.5
Salt	0.2	0.2
Water	18.0	18.0
	Analyzed composition, % DM ^2^	
CP ^3^	31.36	19.20
EE ^4^	9.80	10.04
Ash	6.26	5.32
CF ^5^	0.01	0.14
NFE ^6^	52.57	65.30
Calcium	0.96	1.02
Phosphorus	0.91	0.73
ME, kcal/kg DM ^7^	4109.7	4096.7

^1^ Vitamin and mineral premix supplied per kg of diets: 4375 IU vitamin A; 312.50 IU vitamin D_3_; 31.25 mg vitamin E; 0.06 mg vitamin K; 3.50 mg vitamin B_1_; 3.25 mg vitamin B_2_; 2.50 mg vitamin B_6_; 0.02 mg vitamin B_12_; 7.50 mg Cal-d-pantothenate; 37.50 mg niacin; 0.50 mg folic acid; 0.05 mg biotin; 1250 mg taurine; 55 mg C_4_H_2_FeO_4_; 4.75 mg MnSO_4_; 62.50 mg ZnO; 9.38 mg CuSO_4_; 0.23 mg Na_2_SeO_3_; 1.13 mg KI. ^2^ DM, dry matter; ^3^ CP, crude protein; ^4^ EE, ether extract; ^5^ CF, crude fiber; ^6^ NFE, nitrogen-free extract; ^7^ ME, metabolizable energy.

**Table 2:** Analyzed indispensable amino acid concentrations of the experimental diets and adult maintenance comparison values (% DM, with g/1000 kcal predicted ME in parentheses).

Amino Acid	CP 30% (Analyzed)	CP 18% (Analyzed)	NIAS	AAFCO	NRC RA	FEDIAF 2025
Arginine	1.99 (4.85)	1.14 (2.78)	0.51 (1.28)	0.51 (1.28)	0.35 (0.88)	0.52 (1.30)
Histidine	0.80 (1.93)	0.47 (1.15)	0.19 (0.48)	0.19 (0.48)	0.19 (0.48)	0.23 (0.58)
Isoleucine	1.30 (3.16)	0.75 (1.83)	0.38 (0.95)	0.38 (0.95)	0.38 (0.95)	0.46 (1.15)
Leucine	2.25 (5.47)	1.29 (3.15)	0.68 (1.70)	0.68 (1.70)	0.68 (1.70)	0.82 (2.05)
Lysine	2.25 (5.47)	1.18 (2.88)	0.42 (1.05)	0.63 (1.58)	0.35 (0.88)	0.42 (1.05)
Methionine	0.73 (1.78)	0.50 (1.22)	0.33 (0.83)	0.33 (0.83)	0.33 (0.83)	0.40 (1.00)
Methionine + cystine	1.09 (2.64)	0.81 (1.96)	0.65 (1.63)	0.65 (1.63)	0.65 (1.63)	0.76 (1.91)
Phenylalanine	1.14 (2.76)	0.71 (1.73)	0.45 (1.13)	0.45 (1.13)	0.45 (1.13)	0.54 (1.35)
Phenylalanine + tyrosine	2.02 (4.91)	1.22 (2.98)	0.74 (1.85)	0.74 (1.85)	0.74 (1.85)	0.89 (2.23)
Threonine	1.29 (3.13)	0.74 (1.80)	0.48 (1.20)	0.48 (1.20)	0.43 (1.08)	0.52 (1.30)
Tryptophan	0.32 (0.77)	0.18 (0.44)	0.16 (0.40)	0.16 (0.40)	0.14 (0.35)	0.17 (0.43)
Valine	1.51 (3.68)	0.93 (2.27)	0.49 (1.23)	0.49 (1.23)	0.49 (1.23)	0.59 (1.48)

NIAS and AAFCO values are adult maintenance reference values; NRC RA denotes the NRC recommended allowance; FEDIAF 2025 values are based on a maintenance energy requirement (MER) of 110 kcal ME/kg BW^0.75^/day.

**Table 3:** Body weight and body condition score of adult dogs during the sequential feeding periods.

Item	Week 0	Week 4	Week 8	*p*-Value (0–4)	*p*-Value (4–8)
BW, kg	10.2 ± 0.76	10.2 ± 0.75	10.1 ± 0.72	0.998	0.895
BCS	5.2 ± 0.39	4.7 ± 0.50	4.7 ± 0.45	0.188	1.000

Values are presented as mean ± SEM (*n* = 10). Dogs received the CP 30% formulation during weeks 0–4 and the CP 18% formulation during weeks 4–8. Body weight was analyzed using paired *t*-tests, whereas BCS was analyzed using the Wilcoxon signed-rank test.

**Table 4:** Serum biochemical parameters of adult dogs fed diets containing 30% and 18% crude protein and published canine reference intervals.

Item	CP 30%	CP 18%	*p*-Value	*q*-Value	Published Canine RI *
ALT, U/L ^1^	48.9 ± 5.15	44.5 ± 3.12	0.474	0.576	17–95
AST, U/L ^2^	33.4 ± 2.03	28.5 ± 1.32	0.058	0.180	18–56
ALP, U/L ^3^	63.7 ± 10.24	64.9 ± 7.17	0.925	0.981	7–115
LDH, U/L ^4^	50.0 ± 3.55	36.5 ± 4.91	<0.05	0.166	24–388
CK, U/L ^5^	156.3 ± 6.70	135.8 ± 8.31	0.070	0.180	64–314
GLU, mg/dL ^6^	102.1 ± 2.06	94.2 ± 2.33	<0.05	0.113	68–104
TP, g/dL ^7^	5.5 ± 0.11	5.1 ± 0.19	0.074	0.180	5.5–7.2
ALB, g/dL ^8^	3.6 ± 0.05	3.4 ± 0.12	0.175	0.331	3.2–4.1
TCHO, mg/dL ^9^	196.7 ± 11.27	183.2 ± 11.11	0.405	0.530	136–392
TG, mg/dL ^10^	48.5 ± 3.99	54.0 ± 4.21	0.356	0.504	23–102
Ca, mg/dL ^11^	9.9 ± 0.23	9.4 ± 0.27	0.137	0.291	9.4–11.1
P, mmol/L ^12^	1.3 ± 0.04	1.3 ± 0.05	0.872	0.981	0.87–1.74
AMY, U/L ^13^	633.8 ± 82.00	509.2 ± 61.32	0.240	0.371	322–1310
LIP, U/L ^14^	40.0 ± 8.18	28.3 ± 3.18	0.199	0.338	15–228 ^†^
CREA, mg/dL ^15^	0.7 ± 0.03	0.7 ± 0.03	0.981	0.981	0.6–1.4
Urea, mg/dL	26.2 ± 0.97	17.3 ± 0.67	< 0.001	< 0.001	—
Ammonia, μg/dL	204.8 ± 10.27	84.5 ± 3.44	< 0.001	< 0.001	—

^1^ ALT, alanine aminotransferase; ^2^ AST, aspartate aminotransferase; ^3^ ALP, alkaline phosphatase; ^4^ LDH, lactate dehydrogenase; ^5^ CK, creatine kinase; ^6^ GLU, glucose; ^7^ TP, total protein; ^8^ ALB, albumin; ^9^ TCHO, total cholesterol; ^10^ TG, triglycerides; ^11^ Ca, calcium; ^12^ P, phosphorus; ^13^ AMY, amylase; ^14^ LIP, lipase; ^15^ CREA, creatinine. * RI, reference interval. Values are mean ± SEM (10 dogs); *p*- and *q*-values are from the stated paired/BH procedures. External canine reference intervals are for descriptive comparison (Cornell AHDC,Roche cobas c 501 chemistry analyzer) and may not apply directly to cobas c 111. ^†^ Lipase range is method-dependent. No serum ammonia interval was assigned because the reagent is validated for plasma; an assay-specific urea range was not established.

**Table 5:** Nitrogen balance and excretory characteristics of adult dogs fed diets containing 30% and 18% crude protein.

Item	CP 30%	CP 18%	Paired Difference (95% CI)	*p*-Value	*q*-Value
	Intake characteristics				
Water intake, mL/day	321.4 ± 30.52	272.7 ± 27.46	−48.73 (−68.89 to −28.56)	<0.001	<0.001
Actual feed intake, g/day per dog	220.0	227.0	—	—	—
Dietary N concentration, % as fed	3.98	2.21	—	—	—
Dietary N intake, g N/day	8.76	5.02	—	—	—
	Urinary characteristics				
Urinary pH	6.8 ± 0.07	6.9 ± 0.05	0.059 (−0.137 to 0.256)	0.510	0.613
Urinary specific gravity	1.040 ± 0.004	1.041 ± 0.004	0.00024 (−0.004 to 0.005)	0.902	0.958
Urine output, g/day	213.8 ± 28.84	138.4 ± 15.36	−75.40 (−118.70 to −32.09)	<0.01	<0.01
Urinary N concentration, %	2.9 ± 0.30	2.7 ± 0.28	−0.237 (−0.563 to 0.089)	0.134	0.201
Urinary N excretion, g/day	5.60 ± 0.35	3.34 ± 0.15	−2.252 (−2.984 to −1.520)	<0.001	<0.001
	Fecal characteristics				
Fecal DM output, g/day	12.4 ± 1.42	14.2 ± 0.94	1.799 (−1.231 to 4.829)	0.212	0.283
Fecal N concentration, % DM	5.1 ± 0.09	4.4 ± 0.11	−0.690 (−0.890 to −0.491)	<0.001	<0.001
Fecal N excretion, g/day	0.64 ± 0.07	0.64 ± 0.05	−0.004 (−0.150 to 0.143)	0.958	0.958
	N balance				
Calculated N balance, g N/day	2.52 ± 0.39	1.04 ± 0.14	−1.483 (−2.276 to −0.691)	<0.01	—
N retention, % of intake	28.78 ± 4.45	20.66 ± 2.81	−8.119 (−17.325 to 1.087)	0.077	0.132
Total urinary + fecal N excretion, g/day	6.24 ± 0.39	3.98 ± 0.14	−2.256 (−3.048 to −1.464)	<0.001	<0.001
Total N excretion/BW^0.75^, g/kg BW^0.75^/day	1.125 ± 0.095	0.726 ± 0.048	−0.399 (−0.553 to −0.245)	<0.001	<0.001
Total N excretion, % N intake	71.22 ± 4.45	79.34 ± 2.81	8.119 (−1.087 to 17.325)	0.077	0.132 *

Values are mean ± SEM (*n* = 10). Paired differences are CP 18%–CP 30% with 95% CI. Feed intake was identical to the offered amount because no refusals occurred. The dietary as-fed Kjeldahl N values were 3.98% and 2.21%. N balance is the main comparison (not included in FDR correction); 12 secondary N/excretory outcomes were BH-adjusted. * Total excretion % is exactly 100—N retention % and was not an additional independent hypothesis test; the same *p*/*q* are shown for transparency. BW normalization uses period-end BW. Urine was weighed (g/day).

**Table 6:** Apparent total tract digestibility of nutrients and amino acids in adult dogs fed diets containing 30% and 18% crude protein.

Item	CP 30%	CP 18%	*p*-Value	*q*-Value
	ATTD of analyzed nutrients, %			
DM	93.3 ± 0.75	92.0 ± 0.51	0.195	0.325
CP	92.1 ± 0.96	85.7 ± 1.06	<0.001	<0.01
EE	98.1 ± 0.32	97.8 ± 0.24	0.470	0.588
Ash	60.4 ± 4.25	43.4 ± 4.37	<0.05	<0.05
NFE	97.7 ± 0.46	97.7 ± 0.46	0.952	0.952
	ATTD of amino acids			
Arginine	96.1 ± 0.49	93.3 ± 0.61	<0.05	<0.01
Histidine	93.5 ± 0.68	89.9 ± 0.80	<0.05	<0.01
Isoleucine	95.0 ± 0.58	90.5 ± 0.88	<0.001	<0.01
Leucine	95.5 ± 0.57	91.2 ± 0.84	<0.001	<0.01
Lysine	94.4 ± 0.63	90.0 ± 0.84	<0.01	<0.01
Methionine	94.7 ± 0.71	92.4 ± 0.81	<0.05	<0.05
Cystine	84.2 ± 2.47	81.4 ± 2.14	0.402	0.402
Phenylalanine	94.2 ± 0.75	89.2 ± 1.01	<0.05	<0.01
Tyrosine	94.4 ± 0.73	88.8 ± 1.27	<0.05	<0.01
Threonine	93.9 ± 0.68	88.0 ± 1.11	<0.001	<0.01
Tryptophan	92.7 ± 0.93	85.9 ± 2.19	<0.05	<0.05
Valine	94.6 ± 0.63	89.9 ± 0.96	<0.05	<0.01

Values are mean ± SEM (*n* = 10). Comparisons used paired tests with BH-FDR adjustment in the nutrient and amino acid outcome families. Gross energy and metabolizable energy digestibilities were not experimentally determined and are therefore not included. NFE percentages were independently calculated and happen to round to the same displayed mean and SEM.

## Data Availability

The raw data supporting the conclusions of this article will be made available by the authors on request.

## Supplementary Materials

The following supporting information can be downloaded at: https://www.mdpi.com/article/10.3390/ani16213436/s1. Table S1: Hematological parameters of adult dogs fed diets containing 30% and 18% crude protein; Table S2: Apparent total tract digestibility of nonessential amino acids in adult dogs fed diets containing 30% and 18% crude protein.
