# Supplementary material for: Physiological and Nitrogen Balance Responses of Adult Dogs Fed Diets Containing Different Crude Protein Levels
Source: Animals (Basel). 2026 Oct 30;16(21):3436. doi: 10.3390/ani16213436 (PMC13761580; doi:10.3390/ani16213436)
Supplement: Supplementary file 1 [file animals-16-03436-s001.zip › animals-4527963-supplementary.pdf]

**Supplementary Table S1.** Hematological parameters of adult dogs fed diets containing 30% and 18% crude protein.

| Item | CP 30% | CP 18% | Reference interval | *p*-value | *q*-value |
|---|---|---|---|---|---|
| Leukocytes ($\times 10^3/\mu L$) | | | | | |
| WBC | 9.36 ± 1.07 | 7.72 ± 0.56 | 5.05 – 16.76 | 0.191 | 0.448 |
| NEU | 6.31 ± 1.02 | 4.82 ± 0.37 | 2.95 – 11.64 | 0.187 | 0.448 |
| LYM | 2.38 ± 0.21 | 2.38 ± 0.23 | 1.05 – 5.10 | 0.985 | 0.985 |
| MON | 0.40 ± 0.08 | 0.33 ± 0.02 | 0.16 – 1.12 | 0.360 | 0.675 |
| EOS | 0.24 ± 0.02 | 0.18 ± 0.02 | 0.06 – 1.23 | < 0.05 | 0.293 |
| BAS | 0.02 ± 0.00 | 0.01 ± 0.00 | 0.00 – 0.10 | 0.145 | 0.478 |
| Erythrocytes | | | | | |
| RBC, $\times 10^6/\mu L$ | 7.1 ± 0.17 | 6.9 ± 0.16 | 5.65 – 8.87 | 0.417 | 0.695 |
| HGB, g/dL | 16.8 ± 0.38 | 16.7 ± 0.36 | 13.1 – 20.5 | 0.791 | 0.985 |
| HCT, % | 46.6 ± 1.00 | 45.0 ± 0.89 | 37.0 – 61.7 | 0.245 | 0.525 |
| MCV, fL | 65.6 ± 0.62 | 65.1 ± 0.59 | 61.6 – 73.5 | 0.566 | 0.849 |
| MCHC, g/dL | 36.0 ± 0.08 | 37.0 ± 0.13 | 32.0 – 37.9 | < 0.001 | < 0.001 |
| RDW-CV, % | 13.3 ± 0.14 | 13.2 ± 0.16 | 13.6 – 21.0 | 0.889 | 0.985 |
| Thrombocytes | | | | | |
| PLT, $\times 10^3/\mu L$ | 249.3 ± 26.47 | 248.6 ± 13.54 | 148 – 484 | 0.981 | 0.985 |
| MPV, fL | 10.0 ± 0.28 | 9.9 ± 0.24 | 8.7 – 13.2 | 0.771 | 0.985 |
| PDW | 15.3 ± 0.04 | 15.4 ± 0.08 | 9.1 – 19.4 | 0.070 | 0.350 |

Values are presented as mean ± SEM. Differences between the CP 30% and CP 18% feeding periods were analyzed using a paired t-test. Reference intervals are the manufacturer provided canine reference intervals for the hematology analyzer. CP, crude protein; WBC, white blood cells; NEU, neutrophil; LYM, lymphocytes; MON, monocyte; EOS, eosinophil; BAS, basophils; RBC, red blood cells; HGB, hemoglobin; HCT, hematocrit; MCV, mean corpuscular volume; MCHC, mean corpuscular hemoglobin concentration; RDW-CV, red cell distribution width – coefficient of variation; PLT, platelet; MPV, mean platelet volume; PDW, platelet distribution width.

**Supplementary Table S2.** Apparent total tract digestibility of non-essential amino acids in adult dogs fed diets containing 30% and 18% crude protein.

| Item | CP 30% | CP 18% | *p*-value | *q*-value |
|---|---|---|---|---|
| Aspartic acid, % | 93.2 ± 0.72 | 88.5 ± 1.00 | < 0.001 | < 0.01 |
| Serine, % | 89.8 ± 1.12 | 81.4 ± 1.33 | < 0.001 | < 0.01 |
| Glutamic acid, % | 95.1 ± 0.57 | 91.4 ± 0.79 | < 0.01 | < 0.01 |
| Proline, % | 92.6 ± 0.86 | 87.2 ± 1.26 | < 0.01 | < 0.01 |
| Glycine, % | 93.0 ± 0.74 | 86.1 ± 1.25 | < 0.001 | < 0.01 |
| Alanine, % | 93.7 ± 0.78 | 87.2 ± 1.10 | < 0.001 | < 0.01 |

Values are presented as mean ± SEM. Differences between the CP 30% and CP 18% feeding periods were analyzed using a paired t-test. The *q*-values represent *p*-values adjusted for the false discovery rate (FDR) using the Benjamini-Hochberg procedure within the amino acid outcome family. CP, crude protein; SEM, standard error of the mean.
